# Cold-Pressed Walnut-Oil Adulteration with Edible Oils Detection Using Vis-NIR Spectroscopy

**DOI:** 10.3390/foods14223877

**Published:** 2025-11-13

**Authors:** Georgiana Fediuc, Mariana Spinei, Mircea Oroian

**Affiliations:** 1 Faculty of Food Engineering, Stefan Cel Mare University of Suceava, 13 Universitatii Street, 720229 Suceava, Romania; fediuc.georgiana@outlook.com; 2 Integrated Center for Research, Development and Innovation in Advanced Materials, Nanotechnologies, and Distributed Systems for Fabrication and Control (MANSiD), Stefan Cel Mare University of Suceava, 13 Universitatii Street, 720229 Suceava, Romania; mariana.spinei@fia.usv.ro

**Keywords:** walnut oil, adulteration, UV-Vis-NIR, discrimination

## Abstract

The aim of this study is to evaluate the usefulness of UV-Vis-NIR spectroscopy as a tool for detecting the adulteration of cold-pressed walnut oil and other edible oils (rapeseed, sunflower, and soybean oils) at varying percentages. The spectra were recorded between 200 and 1800 nm, but the analyses focused on 350–1650 nm due to high UV and NIR absorption. Color was determined in CIEL*a*b* coordinates to achieve the differences among the samples. The spectra were submitted to several pre-treatment (none, normalization, SNV, MSC, baseline/detrend, first/second derivative, and 1st-order smoothing) to improve the statistical model’s parameters. The differentiation of the samples was carried out using an unsupervised method (principal component analysis—PCA) and two supervised methods (linear discriminant analysis—LDA and partial least squares linear discriminant analysis—PLS-DA). Partial least squares regression (PLS-R) was used for predicting the degree of adulteration. Separation between the authentic and adulterated samples was visible in the PCA scores plot, primarily along the spectral regions of 420–500 nm (pigment-related absorption band) and 1150–1450 nm (lipid-associated band). PLS-DA was superior to DA for the discrimination of authentic/adulterated samples, with baseline spectra of 350–1650 nm yielding a 100% overall accuracy and near-perfect accuracy with MSC (98.48%). PLS-R was able to predict the adulteration level, depending on the pre-treatment applied.

## 1. Introduction

Walnut oil (WO) has a high market value due to its unique characteristics and laborious production procedures; its unique characteristics are given by a remarkable content of polyunsaturated fatty acids (e.g., linoleic acid and linoleic acid) [1]. Walnut oil is a high-value product due to its nutritional characteristics, as well as its superior organoleptic properties. However, due to its high demand, aroma, pleasant smell, health benefits, high-market value, and high price, this oil is frequently subject to fraudulent adulteration practices, especially with cheaper oils such as sunflower oil. This poses a risk to public health given the differences in chemical composition and nutritional values between genuine and falsified oils [2]. For example, canola oil, hazelnut oil, sunflower oil, soybean oil, and corn oil can be used for adulteration. The adulteration of walnut oil with sunflower oil, rapeseed oil, or soybean oil is difficult to identify because they have similar profiles of polyunsaturated acids as walnut oils [3]; for these reasons, there is a necessity to identify chemical makers, or a spectral fingerprint, for the discrimination of authentic samples from adulterated ones. Adulteration of WO has some negative effects on the reputation of a brand and the producing country in the market and can cause health and safety issues, especially if it is purchased for its nutritional and health benefits [4,5], and may affect the quality and safety of food products.

Detecting adulteration of edible oils has become a priority in the food industry, and in this context, traditional methods such as chromatography or mass spectrometry are often costly and time-consuming. Instead, faster and more affordable analytical techniques, such as UV-Vis spectroscopy and fluorescence analysis [6], have gained popularity due to their ability to provide accurate and efficient results in a short period of time and without complex procedures [7].

Spectroscopic techniques have become increasingly recognized as powerful tools in the food sector. This is due not only to the assessment of oxidative stability and overall quality but also to ensure product authentication. Their effectiveness lies in their high sensitivity, rapid analytical performance, and the ability to analyze samples directly without the need for complex pre-treatments [8,9]. Plant pigments, which are naturally found in walnut kernels, play a significant role in maintaining health. Diets rich in carotenoids provide antioxidant protection and may prevent cardiovascular disease. Carotenoids, along with polyphenols and tocopherols, contribute to the oxidative stability of walnut oils. The natural color of walnut oil is an essential visual attribute that strongly influences the acceptability of the product by consumers [10,11].

UV-Vis spectroscopy analyzes how a material absorbs light in the ultraviolet and visible regions, and this technique is already well established for the detection of adulteration in various types of edible oils. Recent studies have demonstrated that UV-Vis analysis can identify the differences in absorption between pure and adulterated oils by means of a distinct spectral profile generated by the chemical compounds in the oils [7]. UV-Vis and NIR spectroscopy have become convenient methods to identify the adulteration of oils with cheaper ones due to their advantages: they are fast, non-destructive, environmentally friendly, and do not require sample pre-treatment. The overtones and combinations of CH, OH, and NH functional groups present in food samples give rise to absorption in the NIR region. The NIR spectra are characterized by broad and overlapping absorption bands, which makes quantitative analysis difficult, since separating the contribution of a particular functional group from the total signals is not easy to achieve [12].

Over the last years, there have been many studies focused on combing spectroscopic techniques with multivariate analysis to develop rapid methods for the identification of oil adulteration [13]. The chemometric tools applied involved many multivariate analyses, such as principal component analysis, partial least squares regression, and partial least squares discriminant analysis [14,15]. Partial least squares analysis has been proved to be a promising tool for developing statistical models for detecting the adulteration of red fruit oil with coconut oil [15], reaching high determination coefficients (up to 0.99).

Color, another indicator of vegetable-oil quality, plays an essential role, as it directly reflects both compositional attributes and the degree of preservation. Its determination has traditionally been based on standardized colorimetric methods, using dedicated analytical devices, namely, the Konica Minolta colorimeter [16]. To determine the color of the oils, the CIELAB 1976 space was used, giving the best results [10,17]. The colors were evaluated from the chromatic ordinates L*, a*, and b* of the absorption spectrum. The color changes of the analyzed oil samples are directly related to the pigment content and the a* and b* values, which, in turn, are influenced by the degree of adulteration [16,18].

This work aims to explore the combined efficiency of UV-Vis-NIR for the detection and quantification of adulteration of walnut oil with sunflower oil, rapeseed oil, and soybean oil. The objective is to demonstrate how these techniques can be used to highlight the distinct spectral characteristics of each type of oil and to estimate the degree of adulteration, even at low levels of adulteration. Furthermore, this paper attempts to critically present the contribution of UV-Vis-NIR spectroscopy to the ongoing investigation into the quality and authenticity of walnut oil, focusing on the most important studies in this direction. To our knowledge, there are no other studies in the literature that deal with the adulteration of walnut oil with sunflower, rapeseed, and soybean oils.

## 2. Materials and Methods

### 2.1. Materials

Sixteen walnut oil samples were obtained by cold pressing from various walnut sources in Suceava County in 2023. The samples were kept in glass bottles and stored at 4–8 °C. For the falsification of walnut oil, mixtures of walnut oil and sunflower oil, rapeseed oil and soybean oil were prepared in different concentrations. Two types of commercial sunflower oil were used, i.e., rapeseed oil and soybean oil from different manufacturers. Five samples of rapeseed oil were spiked with type I and type II sunflower oils in concentrations of 5%, 10%, 20%, 20%, 30%, 40%, and 50%, resulting in a total of 60 samples. Similarly, the oil samples adulterated with rapeseed oils type I and type II and soybean oils type I and type II were in the number of 60 each. This resulted in 180 samples of adulterated walnut oil.

### 2.2. UV-Vis-NIR Spectroscopy Measurements

The spectra were recorded on a UV-3600 spectrophotometer (Shimadzu Corporation, Kyoto, Japan) in the range 200–1800 nm, using 10 × 10 mm quartz cuvettes. The reference sample was distilled water. Each spectra is the mean of three determinations.

### 2.3. Color Measurement

The color of the samples was measured with a colorimeter (CR-10 Konica Minolta, Tokyo, Japan). All samples were uniformly distributed before measurement. L* described black to white, a* described green (−) to red (+), and b* described blue (−) to yellow (+). Equation (1) was used to calculate the total color change (ΔE). The L_0_*, a_0_*, and b_0_* values were obtained from WO.

(1) $$\Delta E^{*}=\sqrt{{\Delta a^{*}}^{2}+{\Delta b^{*}}^{2}+{\Delta L^{*}}^{2}}$$

### 2.4. Statistical Analysis

The results were analyzed using analysis of variance (ANOVA) with XLSTAT trial version (Lumivero, LLC, Denver, CO, USA). Fisher’s least significant difference (LSD) procedure was applied at a 95% confidence level. Data pre-processing, including normalization, SNV, baseline correction, MSC, detrend, first and second derivatives, and smoothing with a 1st polynomial order (5 smoothing points), was performed using Unscrambler X software version 10.1 (Camo, Oslo, Norway) to reduce noise that could interfere with parameter prediction. The PLS technique was applied to verify whether a linear correlation between the degree of adulteration and the UV-Vis-NIR spectra can be established using the wavelength region under investigation. The UV-Vis-NIR spectra obtained for the walnut oil samples were analyzed in detail at several significant spectral points, covering the visible and near-infrared (350–1650 nm) regions. The absorption values obtained reflect the specific chemical composition of an unrefined walnut oil, and their interpretation allowed for conclusions to be drawn on the degree of processing, stability, and possible source of the oil. The data were analyzed using PLS-DA and DA to develop a suitable model for distinguishing authentic walnut oil samples from adulterated ones. The spectral regions of 500–1650 nm and 350–1650 nm were selected for model development due to their significant influence on classification and their specificity to walnut oil samples and the adulterants (sunflower oil, rapeseed oil, and soybean oil). Before being submitted to PLS-DA/DA, the spectra were pre-treated using various methods, including no treatment, normalization, SNV, baseline correction, MSC, detrend, first and second derivatives, and smoothing with a 1st-polynomial order (5 smoothing points), to enhance the statistical parameters of the classification. The data were divided into two sets—a calibration set (80% of the samples) and a cross-validation set (20% of the samples)—with samples randomly selected by the software. The parameters applied to the PLS-DA algorithm were algorithm-precise, stop conditions at 9, and the following cross-validation method: Jackknife at a confidence interval of 95%. The DA algorithm’s parameters were a stepwise (forward) model, threshold value to enter 0.05, and the following threshold value to remove: 0.1.

## 3. Results and Discussion

### 3.1. UV-Vis-NIR Spectra

The UV-Vis-NIR spectra obtained for the walnut oil samples were analyzed in detail at several significant spectral points, covering the ultraviolet, visible, and near-infrared (200–1800 nm) regions, due to the high absorption of the samples between 200 and 350 nm and 350 and 1800 nm, and the spectra between 350 and 1650 nm were taken into consideration. The absorption values obtained reflect the specific chemical composition of cold-pressed walnut oil, and their interpretation allowed conclusions to be drawn on the degree of processing, stability and possible source of the oil.

**Figure 1:**
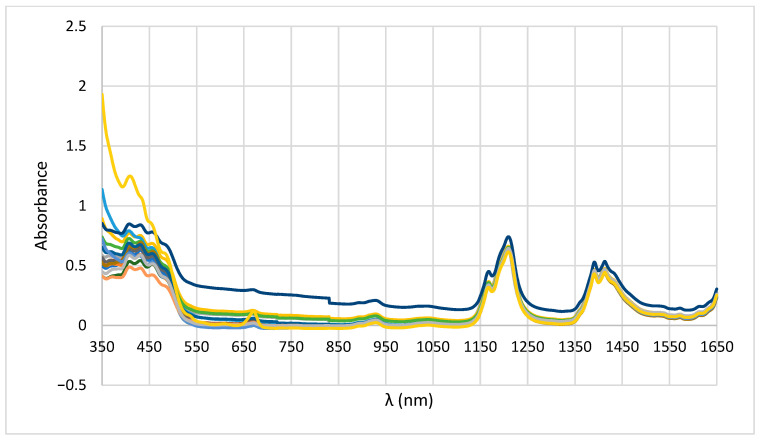
Authentic walnut oil spectra.

Figure 1 shows the spectra of 16 pure walnut oil samples. An absorbance of 0.29 at 652 nm corresponds to chlorophyll *a*, a pigment retained in cold-pressed oils but absent or reduced (<0.10) in refined oils. This confirms that the analyzed sample was unrefined, having a natural pigment composition. Additional absorption at 471 nm also serves as a marker for differentiating genuine oils from blended or counterfeit products [19].

In the NIR region, overtones of C–H and O–H vibrations were observed. An absorbance of 0.72 at 1212 nm reflects a high content of monounsaturated fatty acids, particularly linoleic acid, which is characteristic of walnut oil but lower in refined or polyunsaturated-rich oils such as sunflower [20]. Constant absorbances at 1394 nm (0.52), 1413 nm (0.46), and 1415 nm (0.52) are typical of CH_2_ and CH_3_ groups in fatty acids and indicate a stable lipid structure without degradation [15,21].

#### 3.1.1. Walnut Oil Adulterated with Sunflower Oil

**Figure 2:**
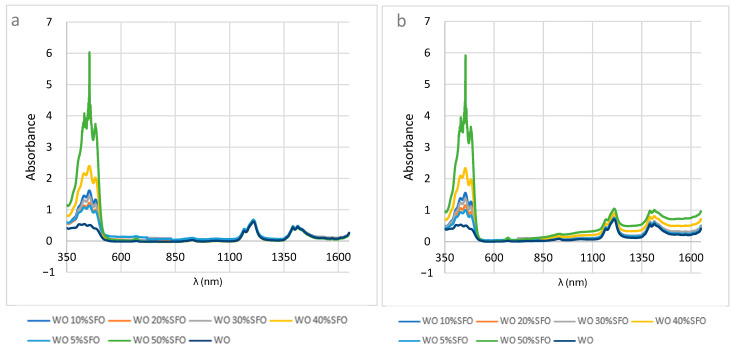

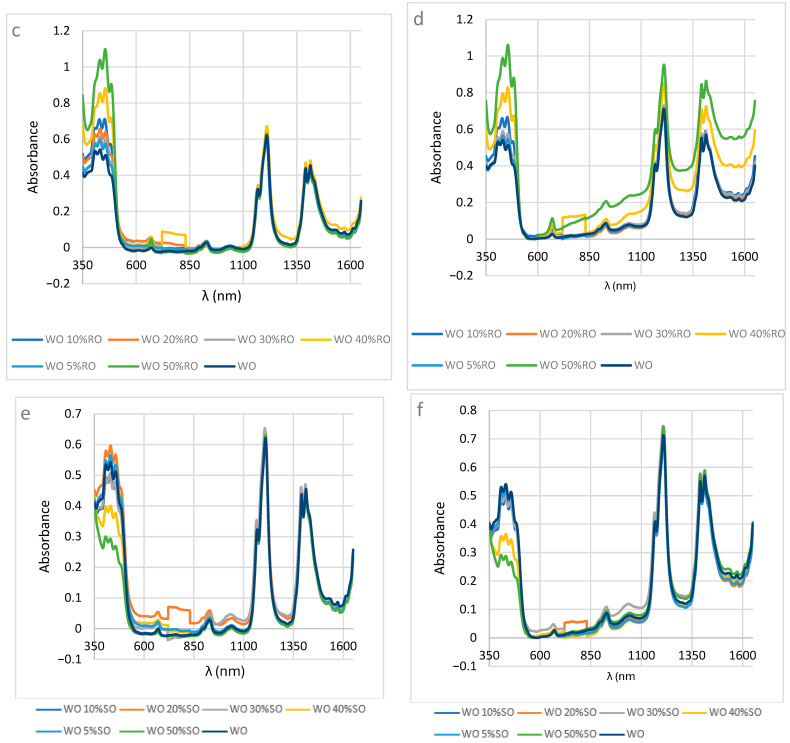
Vis-NIR spectra of walnut oil (WO) adulterated with sunflower oil (SFO)—(**a**) unprocessed spectra and (**b**) baseline corrected spectra; with rapeseed oil—(**c**) unprocessed spectra and (**d**) baseline corrected spectra; and with soybean oil—(**e**) unprocessed spectra and (**f**) baseline corrected spectra.

Figure 2a,b show the spectra of walnut oil (WO) adulterated with sunflower oil (SFO) at 5–50%. Spectral changes were observed mainly in the 350–550 nm and 1150–1450 nm regions. At low adulteration levels (5–10%), the absorbance values at 430–432 nm (0.54–0.56) remained close to those of pure walnut oil (0.53 at 434 nm), indicating that small additions of sunflower oil do not significantly alter pigment-related signals. At higher adulteration (20–40%), the absorptions shifted (417–459 nm, 0.38–0.56), reflecting notable changes in carotenoid content. At 50% adulteration, absorbance at 437 nm dropped to 0.38, showing a significant alteration of the spectrophotometric profile, consistent with dilution by sunflower oil.

#### 3.1.2. Walnut Oil Adulterated with Rapeseed Oil

Figure 2c,d show walnut oil adulterated with rapeseed oil (RO). Pure walnut oil exhibited an absorbance of ~0.51 at 464 nm, whereas the 5% RO sample showed 0.52 at 455 nm, similar to the authentic oil. At 10% adulteration, however, the absorption increased to 0.70 at 430 nm, indicating pigment shifts due to rapeseed oil. Intermediate adulteration levels (20–30%) maintained carotenoid-like absorptions (0.54 at 431–433 nm), but the 40% RO sample showed a marked increase (0.83 at 474 nm), clearly deviating from pure walnut oil. At 50% adulteration, absorbance rose sharply to 1.04, confirming substantial compositional changes and pigment alterations.

#### 3.1.3. Walnut Oil Adulterated with Soybean Oil

Figure 2e,f illustrate walnut oil adulterated with soybean oil (SO). Pure walnut oil showed an absorbance of ~0.50 at 438 nm, characteristic of carotenoids. The 5% SO sample displayed an intense absorbance of 1.00 at 455 nm, reflecting a strong pigment signal possibly enhanced by soybean oil. At 10% adulteration, absorbances at 451 nm (0.50) and 1198 nm (0.60) suggested reduced natural pigments and altered lipid composition. At 30% SO, absorptions were observed at 458 nm (0.98) and 1415 nm (0.56), indicating carotenoid dilution and changes in fatty acid profiles. The 40% SO sample showed very strong absorption at 460 nm (2.2), possibly reflecting pigment masking. Finally, the 50% SO sample exhibited unusually intense absorption at 455 nm (5.88), accompanied by changes in the NIR region (1218 nm, 0.68), indicating significant adulteration and lipid profile alteration.

### 3.2. Walnut Oil Color

Cold-pressed walnut oil is a valuable source of polyunsaturated fatty acids (PUFAs), including linoleic and α-linolenic acids, as well as monounsaturated fatty acids (MUFAs) such as palmitoleic, oleic, and erucic acids. In addition, it contains bioactive compounds, such as chlorophylls, tocopherols, and vitamins K and E. Cold-pressed walnut oils are typically light yellow with greenish hues, a characteristic color primarily attributed to lipophilic pigments, including carotenoids and chlorophylls. Walnut oil contains a wide spectrum of these pigments, ranging from β-carotene, violaxanthin, neoxanthin, and lutein to chlorophylls a and b, pheophytins a and b, and other minor derivatives [22].

The color parameters of walnut oil are typically within the following ranges: L* = 20.39–21.12; a* = –0.5 to –1.3; b* = 4.33–5.66; C* = 7.91–10.72; and h* = 95.88–102.03 (Table 1). When sunflower oil is added at levels between 5% and 50%, the brightness (L*) and chromaticity (C*) decrease proportionally, while the hue (h*) increases. As a result, the oils analyzed in this study exhibited a characteristic yellow–green color. From a food application perspective, a clear, light-yellow–green color is desirable.

The obtained results show that the chromatic parameters of walnut and adulterated samples are generally close, though the brightness decreases as the proportion of sunflower oil increases. Both walnut and sunflower oils tend toward the yellow–red region of the color spectrum [23]. The quantified color difference (∆E*) revealed only small variations among the samples. The literature indicates that a ∆E* value ≤ 1.5 does not represent a significant change, and such products are considered acceptable. However, in this study, oils adulterated with ≥5% sunflower oil exceeded this threshold, making them unacceptable from a quality standpoint.

Regarding the whiteness (WI) and yellowness indices (YI), previous studies confirm a strong inverse correlation: as WI increases, YI must decrease for the product to maintain acceptable quality [20].

**Table 1:** Analysis of variance of color parameters of walnut oils adulterated with sunflower, rapeseed, and soybean oils.

**Parameter**	**Adulteration with sunflower oil (%)**							***F*-value**
	0	5	10	20	30	40	50	
L*	20.84 (0.291) ^b^	19.16 (1.26) ^a^	19.19 (1.73) ^ab^	18.97 (0.67) ^a^	19.19 (0.80) ^a^	19.13 (0.92) ^a^	19.15 (0.80) ^a^	4.918 ^ns^
a*	−0.99 (0.29) ^a^	−1.17 (0.25) ^a^	−1.21 (0.15) ^a^	−1.14 (0.10) ^a^	−1.22 (0.11) ^a^	−1.26 (0.18) ^a^	−1.19 (0.18) ^a^	1.62 ^ns^
b*	5.22 (0.52) ^b^	3.23 (0.14) ^a^	3.19 (1.07) ^a^	2.86 (0.68) ^a^	3.05 (0.68) ^a^	2.83 (0.86) ^a^	2.83 (0.77) ^a^	4.55 **
C*	9.8 (1.10) ^b^	5.59 (1.45) ^a^	6.00 (1.88) ^a^	5.49 (1.19) ^a^	5.86 (1.19) ^a^	5.49 (1.54) ^a^	5.48 (1.41) ^a^	6.53 ***
h*	99.26 (2.20) ^a^	110.08 (5.65) ^b^	110.76 (4.76) ^b^	111.36 (4.75) ^b^	111.09 (4.20) ^b^	113.44 (3.74) ^b^	113.26 (3.33) ^b^	7.06 ***
ΔE*	0 (0) ^a^	2.98 (1.38) ^b^	2.98 (1.12) ^b^	3.08 (1.25) ^b^	2.83 (1.20) ^b^	3.08 (1.23) ^b^	3.02 (1.20) ^b^	4.89 ***
WI	7.2 (0.42) ^a^	9.01 (0.79) ^b^	9.28 (0.31) ^b^	9.2 (0.51) ^b^	9.28 (0.42) ^b^	9.58 (0.19) ^b^	9.59 (0.22) ^b^	18.59 ***
YI	35.78 (3.19) ^b^	23.42 (8.8) ^a^	22.94 (5.51) ^a^	21.40 (4.47) ^a^	22.58 (4.28) ^a^	20.91 (5.24) ^a^	20.92 (4.81) ^a^	4.97 ***
	**Adulteration with rapeseed** **oil (%)**							*F*-value
	0	5	10	20	30	40	50	
L*	20.84 (0.29) ^b^	19.16 (0.56) ^a^	19.19 (0.74) ^a^	19.14 (0.73) ^a^	19.18 (0.72) ^a^	19.07 (0.67) ^a^	19.10 (0.71) ^a^	4.91 ***
a*	−0.99 (0.299) ^a^	−1.14 (0.25) ^a^	−1.09 (0.13) ^a^	−1.01 (0.15) ^a^	−0.96 (0.14) ^a^	−0.96 (0.12) ^a^	−0.97 (0.12) ^a^	2.05 ^ns^
b*	5.22 (0.523) ^b^	3.04 (0.55) ^a^	3.11 (0.73) ^a^	3.22 (0.84) ^a^	3.36 (0.75) ^a^	3.13 (0.75) ^a^	3.26 (0.64) ^a^	6.59 ***
C*	9.8 (1.10) ^b^	5.7 9(1.005) ^a^	5.87 (1.35) ^a^	6.08 (0.52) ^a^	6.3 (1.38) ^a^	5.88 (1.39) ^a^	6.09 (1.17) ^a^	6.69 ***
h*	99.26 (2.20) ^a^	109.59 (3.38) ^b^	109.11 (4.49) ^b^	107.01 (4.58) ^b^	105.34 (43.61) ^b^	106.43 (2.87) ^b^	105.71 (2.24) ^b^	5.98 ***
ΔE*	0.52 (0.61) ^a^	3.19 (0.74) ^b^	3.13 (0.97) ^b^	3.06 (1.07) ^b^	2.93 (1.02) ^b^	3.17 (0.98) ^b^	3.07 (0.87) ^b^	5.94 ***
WI	7.2 (0.42) ^a^	9.09 (0.42) ^b^	8.92 (0.61) ^b^	8.58 (0.74) ^b^	8.33 (0.56) ^b^	8.54 (0.36) ^b^	8.43 (0.38) ^b^	8.38 ***
YI	35.78 (3.19) ^b^	232.61 (3.66) ^a^	23 (4.69) ^a^	23.87 (5.43) ^a^	24.85 (4.68) ^a^	23.31 (4.86) ^a^	24.27 (4.15) ^a^	5.79 ***
	**Adulteration with soybean oil (%)**							*F*-value
	0	5	10	20	30	40	50	
L*	20.84 (0.291) ^b^	19.15 (0.565) ^a^	18.83 (0.90) ^a^	18.88 (0.96) ^a^	19.09 (0.82) ^a^	19.05 (1.03) ^a^	18.91 (0.93) ^a^	3.21 ^ns^
a*	−0.99 (0.29) ^ab^	−1.09 (0.15) ^a^	−1.06 (0.09) ^a^	−0.95 (0.07) ^a^	−0.89 (0.17) ^a^	−0.86 (0.12) ^a^	−0.77 (0.10) ^a^	5.39 ***
b*	5.22 (0.52) ^b^	3.15 (0.80) ^a^	2.86 (0.52) ^ab^	2.99 (0.67) ^abc^	3.14 (0.90) ^abc^	3.06 (1.18) ^bc^	2.88 (0.96) ^c^	6.06 ***
C*	9.8 (1.10) ^b^	5.94 (1.46) ^a^	5.44 (0.90) ^a^	5.64 (1.17) ^a^	5.88 (1.66) ^a^	5.74 (2.13) ^a^	5.37 (1.74) ^a^	5.72 ***
h*	99.26 (2.20) ^a^	109.06 (4.87) ^b^	109.63 (3.09) ^b^	107.14 (3.69) ^b^	105.54 (4.82) ^ab^	106.42 (5.84) ^b^	105.34 (5.37) ^ab^	4.48 **
ΔE*	0.52 (0.61) ^a^	3.16 (1.18) ^b^	3.54 (0.96) ^b^	3.42 (1.09) ^b^	3.16 (1.18) ^b^	3.26 (1.52) ^b^	3.49 (1.29) ^b^	3.57 **
WI	7.2 (0.42) ^a^	8.85 (0.46) ^b^	8.95 (0.31) ^b^	8.57 (0.36) ^b^	8.36 (0.65) ^b^	8.37 (0.88) ^b^	8.29 (0.72) ^b^	5.91 ***
YI	35.78 (3.19) ^b^	23.29 (4.99) ^a^	21.57 (3.12) ^a^	22.49 (4.06) ^a^	23.29 (5.75) ^a^	22.61 (7.51) ^a^	21.52 (6.10) ^a^	4.99 ***

Mean values and the standard deviation are shown in brackets. ^ns^ *p* > 0.05, ** *p* < 0.001, and *** *p* < 0.0001. ^a–c^ Different letters in the same row indicate significant differences a samples (*p* < 0.0001) according to the LSD test with α = 0.05. L*—brightness; a*—green (−) to red (+); b*—blue (−) to yellow (+). C*—chromaticity; h*—hue angle; ΔE*—color change; WI—whiteness index; YI—yellowness index.

### 3.3. Walnut Oil Classification Using Statistical Analysis

#### 3.3.1. Unsupervised Method—Principal Component Analysis (PCA)

**Figure 3:**
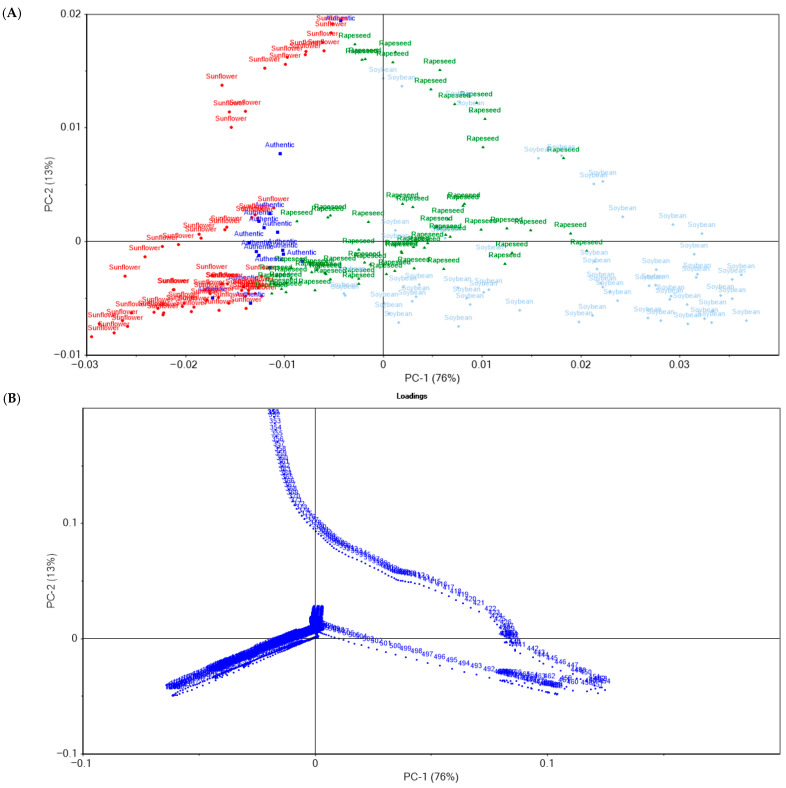
Principal component analysis: (**A**) principal component scores of the walnut oil samples adulterated with sunflower oil, soybean oil, and rapeseed oil; (**B**) principal component loadings.

The principal component analysis (PCA) of the spectral data for walnut oil and its adulterated variants demonstrated a clear differentiation between authentic samples and those containing sunflower, rapeseed, or soybean oil, using the 350–1650 nm spectra, with baseline pre-treatment applied prior to the PCA. The first two principal components (PC1 and PC2) accounted for 89% of the total variance in the dataset, with PC1 explaining 76% and PC2 13%. These results indicate that the majority of the relevant variation was captured by the first two components, rendering the two-dimensional PCA representation adequate for describing the underlying spectral distinctions. As shown in Figure 3A, the authentic walnut oil samples are positioned near the origin of the coordinate axes, suggesting a relatively homogeneous spectral profile. In contrast, the adulterated samples are distributed along the principal component axes, reflecting compositional differences induced by the addition of other oils. Samples adulterated with sunflower oil are displaced toward the positive side of PC2, implying spectral modifications associated with sunflower oil constituents. Rapeseed oil–adulterated samples are distributed along the positive side of PC1, indicating that PC1 captures the main source of variation between authentic and rapeseed-adulterated samples. Soybean oil–adulterated samples occupy the lower-right quadrant, exhibiting a negative correlation with PC2 and a positive shift along PC1, consistent with their distinct compositional influence relative to sunflower- and rapeseed-based adulterations. The loadings presented in Figure 3B show that wavelengths in the 420–500 nm region contributed most strongly to PC1. This region corresponds to pigment-related absorption bands primarily associated with carotenoids and chlorophyll derivatives, which are key factors in distinguishing authentic walnut oil from adulterated samples. Sunflower-oil adulteration correlated with the 450–480 nm range, dominated by carotenoid absorption, consistent with the higher carotenoid content characteristic of sunflower oil. Rapeseed-oil adulteration was associated with the 420–450 nm range, corresponding to chlorophyll-related pigments, typically more abundant in rapeseed oil than in walnut oil. Soybean-oil adulteration exhibited a distinct association with the 350–400 nm region, attributed to aromatic compounds and tocopherols that are characteristic of soybean oil.

These findings show that PCA not only distinguishes authentic walnut oil from adulterated samples but also identifies the type of adulterant through its spectral signature. Authentic oils clustered near the origin, and they lacked pigment and aromatic deviations. Sunflower adulteration shifted samples toward carotenoid absorption, rapeseed reflected chlorophyll features, and soybean correlated with tocopherol and aromatic absorptions. The combined scores and loadings plots link the chemical nature of adulterants to their spectral effects. PC1 is the main axis separating authentic from adulterated oils, while PC2 differentiates adulterant types, confirming PCA’s strong potential for detecting and characterizing walnut-oil adulteration.

#### 3.3.2. Supervised Methods—Discriminant Analysis (DA) vs. Partial Least Squares Linear Discriminant Analysis (PLS-LDA)

The classification of authentic and adulterated walnut oils was performed using two supervised methods—discriminant analysis (DA) and partial least squares linear discriminant analysis (PLS-LDA)—to identify the most suitable model (Table 2 and Supplementary Table S1). The dataset consisted of authentic samples (16) and samples adulterated with sunflower oil (60), rapeseed oil (60), and soybean oil (60). Spectral data were analyzed in the following two wavelength ranges: 350–1650 nm and 500–1650 nm. Several pre-treatment methods were evaluated, including no treatment, normalization, first derivative, second derivative, baseline correction, standard normal variate (SNV), multiplicative scatter correction (MSC), and first-order polynomial smoothing with five smoothing points. For each dataset, samples were randomly divided into calibration (66%) and validation (33%) sets.

The classification performance differed across oil types and pre-treatment methods. Authentic walnut oil was consistently identified with very high accuracy. Several pre-treatments—normalized spectra (350–1650 nm), SNV (350–1650 nm), baseline correction (350–1650 nm), detrending (350–1650 nm), and smoothing (350–1650 nm)—achieved 100% correct classification, confirming the reliable distinction between authentic and adulterated samples. For sunflower-oil adulteration, the highest accuracy was obtained using normalized spectra (350–1650 nm) with discriminant analysis (DA), yielding 89.29% correct classification. Smoothing in the same range led to a similar outcome (90.91%), showing that sunflower adulteration can be detected with relatively high precision. Rapeseed-oil adulteration was more difficult to identify. The best performance reached 77.27% using SNV spectra (500–1650 nm, DA). Although some pre-treatments produced accuracies between 75% and 88%, the results were less consistent, indicating weaker separability compared to other oils. Soybean-oil adulteration showed the most accurate detection. Using SNV spectra (500–1650 nm, DA) resulted in a 100% correct classification, while smoothing (350–1650 nm) achieved 90.48%. These outcomes indicate that soybean adulteration caused the most distinct spectral variations, enabling precise and consistent classification.

Overall, the models displayed clear differences in performance depending on the adulterant type. Soybean adulteration was the most reliably detected, followed by authentic walnut oil, both reaching near-perfect accuracy. Sunflower adulteration showed moderate to high accuracy (89–91%), whereas rapeseed oil remained the most challenging (70–77%). Across all pre-treatment approaches, normalized spectra (350–1650 nm, DA) yielded the highest overall validation accuracy (93.28%). Several other treatments—MSC (500–1650 nm), smoothing (350–1650 nm), original spectra (500–1650 nm), detrend (500–1650 nm), and SNV applied to both spectral ranges—also produced strong and consistent results around 88%. These findings confirm the robustness of discriminant analysis, especially when combined with optimized spectral pre-processing techniques.

Zaukuu et al. used UV-IDS-NIR spectroscopy for detection and quantification of groundnut-oil adulteration with palm olein, and the LDA models were able to distinguish between pure groundnut oil and adulterated samples, and the average cross-validation values were between 62.14 and 92.61% [21]. UV-Vis-NIR absorption coupled with LDA were used for the identification of extra-virgin-oil adulteration with pomace, soybean, corn, and sunflower oils, at various concentrations and the fused dataset, the results concerning the discrimination between pure and adulterated olive oils were found to exceed 99% [17]. UV-Vis coupled with PLS-DA were the best model compared with the other techniques (e.g., FT-IR, Raman, and GC-MS) in the classification of different groups as authentic virgin olive oils, adulterants (safflower oil, corn oil, soybean oil, canola oil, sunflower oil, and sesame oil), or specific adulterated olive oils [23].

#### 3.3.3. Partial Least Squares Regression Correlation of UV-Vis-NIR Spectra with the Degree of Adulteration

The partial least squares regression (PLS-R) models developed in this study were employed to predict the degree of adulteration in walnut oil samples using UV-Vis-NIR spectral data. To optimize model performance, the spectral data were subjected to a series of pre-processing techniques aimed at reducing noise and minimizing background interference, both of which are known to markedly affect predictive accuracy. The following pre-treatment methods were evaluated: no treatment, normalization, first derivative, second derivative, baseline correction, standard normal variate (SNV), multiplicative scatter correction (MSC), and first-order polynomial smoothing with five smoothing points.

Model performance was assessed using four experimental datasets: (a) walnut oil adulterated with sunflower oil (16 authentic samples and 61 adulterated samples containing 5–50% sunflower oil), (b) walnut oil adulterated with rapeseed oil (16 authentic samples and 61 adulterated samples), (c) walnut oil adulterated with soybean oil (16 authentic samples and 61 adulterated samples), and (d) a combined dataset comprising all adulteration types (16 authentic samples and 180 adulterated samples).

**Figure 4:**
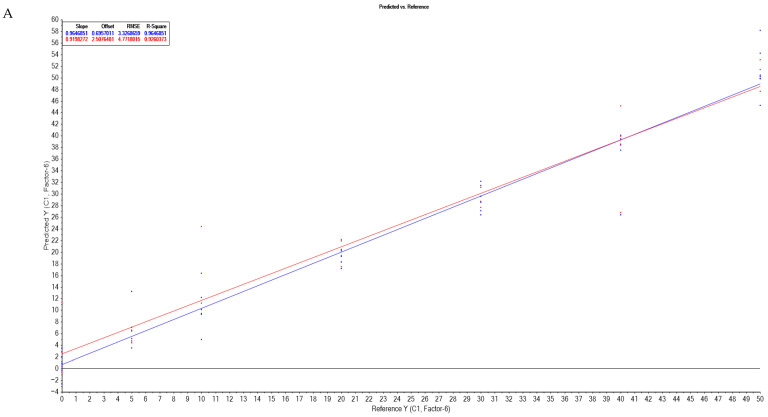

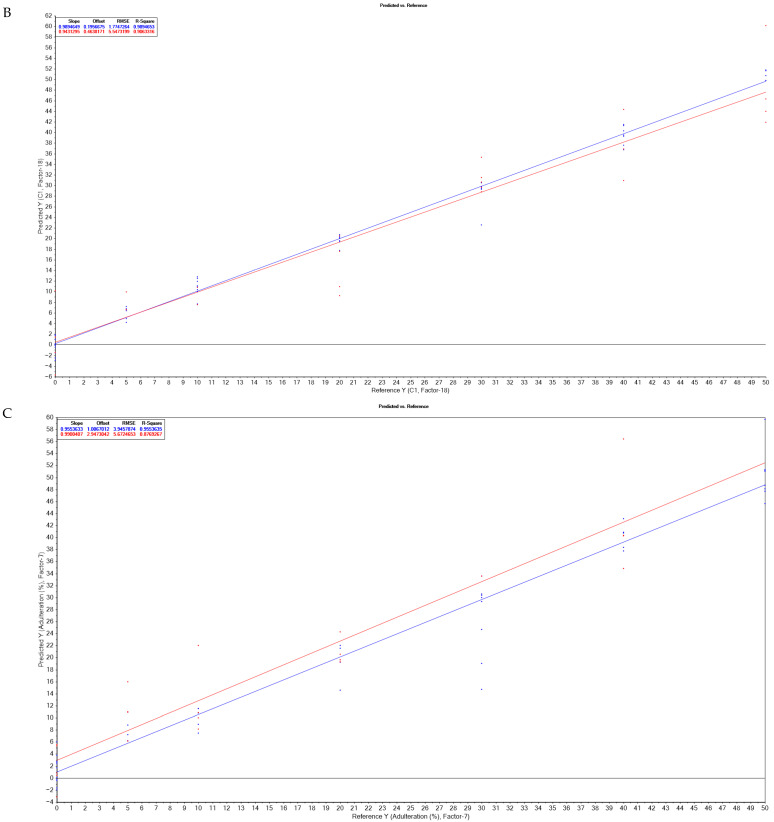

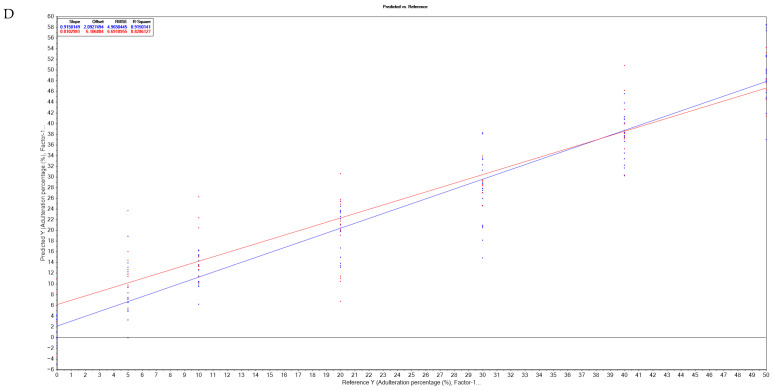
Partial least squares regression (PLS-R) for predicting the degree of adulteration: (**A**) samples adulterated with soybean oils; (**B**) samples adulterated with sunflower oils; (**C**) samples adulterated with rapeseed oils; (**D**) samples adulterated with all the oils.

Spectral data were analyzed in two ranges, 350–1650 nm and 500–1650 nm. For each dataset, samples were randomly divided into calibration (66%) and validation (33%) sets. Model suitability was determined using the coefficient of regression, slope, offset, and root mean square error (RMSE). The statistical performance parameters of the PLS-R models are summarized in Table 3 and Table S2. The results indicate that the most effective pre-treatment method varied according to the adulterant, as follows: sunflower-oil adulteration—original spectra (RMSEC = 1.774, RMSEV = 5.547); soybean-oil adulteration—second derivative (RMSEC = 3.332, RMSEV = 4.471); rapeseed-oil adulteration—first derivative (RMSEC = 3.945, RMSEV = 5.672); and all of the oils—detrend pre-treatment (RMSEC = 4.965, RMSEV = 6.691). The correlation between the reference and predicted adulteration values obtained from the PLS-R models is illustrated in Figure 4. Zaukuu et al. used Vis-NIR spectroscopy for the discrimination of sesame oil and rapeseed oil adulterated with soybean oil, and they observed that MSC pre-treatment of spectra coupled with PLS-R can predict the degree of adulteration (R^2^ higher than 0.97) [21]. Tarhan et al. used UV-Vis spectroscopy of adulteration of sunflower oil with low level of safflower oil and reached lower statistical values than our study for predicting the degree of adulteration with PLS model (R^2^ ranged between n.d. and 0.99) [23].

## 4. Conclusions

Vis-NIR spectroscopy is a fast, non-destructive, solvent-free, practical, and easy-to-handle method for walnut-oil-adulteration detection with sunflower, rapeseed, and soybean oils at varying percentages. The discriminant analysis (DA) applied to the SNV spectra in the 350–1650 nm range achieved the best overall validation accuracy among DA models (86.36%). Detection performance varied depending on the adulterant type, with soybean and rapeseed oils showing higher classification rates (87–95%) compared to sunflower oil (80%). These results indicate that DA provided reliable discrimination for most adulterants, though additional pre-processing optimization would be required to improve performance, particularly for sunflower oil samples. The PLS-R was able to predict the adulteration level. Principal component analysis (PCA) differentiated authentic from adulterated samples primarily through spectral variations in the 420–500 nm region (associated with pigments) and the 1150–1450 nm region (associated with lipids). The concordance between pigment-associated visible spectral features, lipid-related NIR bands, and CIEL*a*b* colorimetric responses (with ΔE* values exceeding the level at which color differences become noticeable to the human eye, starting from 5% sunflower adulteration) shows clearly that both spectral and color measurements can reliably track changes in composition.

## Figures and Tables

**Table 2:** Statistical parameters of the PLS-DA and DA discrimination of authentic and adulterated walnut oil using the Vis-NIR spectra with different pre-treatments.

Pre-Treatment	Model	Step	Samples (%)				Total
			Authentic	Sunflower	Rapeseed	Soybean	
Baseline spectra 350–1650 nm	PLS-DA	Calibration	100.00	100.00	100.00	100.00	100.00
		Validation	100.00	100.00	100.00	100.00	100.00
Detrend spectra 500–1650 nm	DA	Calibration	91.67	77.50	72.22	71.43	78.20
		Validation	75.00	90.00	70.83	61.11	74.24
SNV spectra 350–1650 nm	DA	Calibration	100.00	94.29	95.00	97.73	96.75
		Validation	80.00	80.00	95.00	87.50	86.36
MSC spectra 500–1650 nm	PLS-DA	Calibration	100.00	100.00	100.00	100.00	100.00
		Validation	100.00	94.74	100.00	100.00	98.48

**Table 3:** Regression parameters of the calibration and validation procedures calculated for the Vis-NIR spectra data submitted to partial least squares regression analysis (PLS-R) for predicting the degree of adulteration of the walnut oil samples.

Adulterant	Pre-Treatment	No. Factor	Calibration				Cross-Validation			
			Slope	Offset	RMSE	R^2^	Slope	Offset	RMSE	R^2^
All the samples 500–1650 nm	Detrend	17	0.915	2.0927	4.965	0.915	0.810	6.106	6.691	0.828
Rapeseed oils 350–1650 nm	1st derivate	7	0.955	1.006	3.945	0.955	0.990	2.947	5.672	0.876
Soybean oils 500–1650 nm	2nd derivate	6	0.964	0.695	3.332	0.964	0.919	2.507	4.771	0.926
Sunflower oils 350–1650 nm	No pre-treatment	18	0.989	0.195	1.774	0.989	0.943	0.463	5.547	0.906

## Data Availability

The original contributions presented in this study are included in the article/Supplementary Materials. Further inquiries can be directed to the corresponding author.

## Supplementary Materials

The following supporting information can be downloaded at: https://www.mdpi.com/article/10.3390/foods14223877/s1, Table S1: Statistical parameters of the PLS-DA and DA discriminations of authentic and adulterated walnut oil using the Vis-NIR spectra with different pre-treatments; Table S2: Regression parameters of the calibration and validation procedures calculated for the VIS-NIR spectral data submitted to partial least squares regression analysis (PLS-R) for predicting the degree of adulteration of the walnut oil samples.
