# Supplementary material for: Cold-Pressed Walnut-Oil Adulteration with Edible Oils Detection Using Vis-NIR Spectroscopy
Source: Foods. 2025 Nov 13;14(22):3877. doi: 10.3390/foods14223877 (PMC12651916; doi:10.3390/foods14223877)
Supplement: Supplementary file 1 [file foods-14-03877-s001.zip › foods-3948066-supplementary.pdf]

Table S1. Statistical parameters of the PLS-DA and DA discriminations of authentic and adulterated walnut oil using the Vis-NIR spectra with different pre-treatments.

**Pre-treatment**	**Model**	**Step**	Samples (%)				Total
			Authentic	Sunflower	Rapeseed	Soybean	
**Original spectra 350-1650 nm**	**DA**	**Calibration**	90.00	86.84	72.50	73.81	80.79
		Validation	83.33	77.27	70.00	83.33	77.27
	PLS-DA	Calibration	100.00	100.00	100.00	93.18	97.69
		Validation	100.00	100.00	100.00	87.50	96.97
Original spectra 500-1650 nm	DA	Calibration	100.00	83.33	68.29	68.29	79.98
		Validation	100.00	79.19	89.47	63.16	78.79
	PLS-DA	Calibration	88.89	100.00	100.00	95.12	97.69
		Validation	57.14	100.00	100.00	89.47	92.42
Normalized spectra 350-1650 nm	DA	Calibration	100.00	86.84	81.08	88.64	89.14
		Validation	100.00	72.73	73.91	75.00	75.76
	PLS-DA	Calibration	100.00	97.50	100.00	94.87	97.69
		Validation	100.00	100.00	100.00	90.48	96.97
Normalized spectra 500-1650 nm	DA	Calibration	100.00	72.50	82.05	85.37	84.98
		Validation	83.33	65.00	76.19	68.42	71.21
	PLS-DA	Calibration	100.00	100.00	100.00	97.73	99.23
		Validation	100.00	92.31	100.00	100.00	96.97
Baseline spectra 350-1650 nm	DA	Calibration	87.50	68.42	78.26	73.68	76.97
		Validation	62.50	81.82	64.29	68.19	71.21
	PLS-DA	Calibration	100.00	100.00	100.00	100.00	100.00
		Validation	100.00	100.00	100.00	100.00	100.00
Baseline spectra 500-1650 nm	DA	Calibration	77.78	76.19	70.00	61.54	71.38
		Validation	85.71	55.56	65.00	76.19	68.18
	PLS-DA	Calibration	100.00	100.00	100.00	86.11	96.15
		Validation	100.00	100.00	100.00	75.00	90.91
1st-derivate spectra 350-1650 nm	DA	Calibration	100.00	80.00	84.09	85.71	87.45
		Validation	71.43	76.00	68.75	55.56	68.18
	PLS-DA	Calibration	100.00	100.00	100.00	100.00	100.00
		Validation	100.00	100.00	100.00	100.00	100.00
1st-derivate spectra 500-1650 nm	DA	Calibration	100.00	80.49	75.00	70.00	81.37
		Validation	85.71	73.68	70.00	50.00	66.67
	PLS-DA	Calibration	100.00	100.00	100.00	97.50	99.23
		Validation	100.00	100.00	100.00	95.00	98.48
2nd-derivate spectra 350-1650 nm	DA	Calibration	100.00	100.00	100.00	100.00	100.00
		Validation	50.00	40.91	75.00	59.09	57.58
	PLS-DA	Calibration	90.91	78.05	57.50	84.21	74.62
		Validation	100.00	68.42	45.00	86.36	69.70
2nd derivate spectra 500-1650 nm	DA	Calibration	100.00	100.00	100.00	100.00	100.00
		Validation	83/33	64.00	33.33	13.04	42.42
	PLS-DA	Calibration	90.91	66.67	86.33	87.80	80.77
		Validation	100.00	87.50	83.33	78.95	84.85
	DA	Calibration	90.00	65.85	73.68	70.73	75.07

Detrend spectra 350-1650 nm		Validation	66.67	68.42	81.82	52.63	68.18
	PLS-DA	Calibration	100.00	100.00	100.00	100.00	100.00
		Validation	100.00	100.00	100.00	100.00	100.00
Detrend spectra 500-1650 nm	DA	Calibration	91.67	77.50	72.22	71.43	78.20
		Validation	75.00	90.00	70.83	61.11	74.24
	PLS-DA	Calibration	100.00	100.00	100.00	95.00	98.46
		Validation	100.00	95.24	100.00	90.00	95.45
SNV spectra 350-1650 nm	DA	Calibration	100.00	94.29	95.00	97.73	96.75
		Validation	80.00	80.00	95.00	87.50	86.36
	PLS-DA	Calibration	100.00	100.00	100.00	100.00	100.00
		Validation	100.00	95.24	100.00	100.00	98.48
SNV spectra 500-1650 nm	DA	Calibration	88.89	82.93	92.50	82.50	86.70
		Validation	85.71	52.63	90.00	90.00	78.79
	PLS-DA	Calibration	100.00	94.74	100.00	97.62	97.69
		Validation	100.00	90.91	100.00	100.00	96.97
MSC spectra 350-1650 nm	DA	Calibration	92.31	91.67	93.33	97.22	93.63
		Validation	100.00	79.19	86.67	87.50	84.85
	PLS-DA	Calibration	100.00	97.30	100.00	94.59	97.69
		Validation	100.00	100.00	100.00	96.55	98.48
MSC spectra 500-1650 nm	DA	Calibration	100.00	89.47	88.10	89.47	91.76
		Validation	75.00	90.91	77.78	81.82	83.33
	PLS-DA	Calibration	100.00	100.00	100.00	100.00	100.00
		Validation	100.00	94.74	100.00	100.00	98.48
Smoothing spectra 350-1650 nm	DA	Calibration	100.00	87.50	80.56	92.68	90.18
		Validation	100.00	95.00	58.33	94.74	81.82
	PLS-DA	Calibration	100.00	100.00	100.00	84.62	95.38
		Validation	100.00	100.00	100.00	80.95	93.94
Smoothing spectra 500-1650 nm	DA	Calibration	70.00	70.27	65.12	70.00	68.85
		Validation	56.67	78.26	64.71	50.00	60.61
	PLS-DA	Calibration	100.00	100.00	100.00	91.89	97.69
		Validation	100.00	100.00	100.00	86.96	95.45

Table S2. Regression parameters of the calibration and validation procedures calculated for the VIS-NIR spectral data submitted to partial least squares regression analysis (PLS-R) for predicting the degree of adulteration of the walnut oil samples.

**Adulterant**	**Pre-treatment**	No. factor	Calibration				Cross-Validation			
			Slope	Offset	RMSE	R^2	Slope	Offset	RMSE	R^2
**All samples 350-1650 nm**	**No pre-treatment**	6	0.540	11.312	11.543	0.540	0.621	10.937	12.117	0.438
	Normalized	4	0.443	13.698	12.702	0.443	0.493	12.935	12.497	0.402
	1st derivate	8	0.408	14.558	13.095	0.408	0.310	15.268	13.981	0.251
	2nd derivate	8	0.408	14.558	13.095	0.408	0.310	15.268	13.981	0.251
	Baseline	6	0.564	10.712	11.233	0.564	0.559	11.399	11.049	0.532
	SNV	10	0.644	8.755	10.155	0.644	0.566	11.991	11.487	0.494
	Detrend	5	0.437	13.853	12.774	0.437	0.493	13.603	12.671	0.385
	MSC	4	0.484	12.703	12.232	0.484	0.520	12.271	12.133	0.436

	Smoothing	6	0.572	10.535	11.140	0.572	0.607	11.094	11.892	0.458
All samples 500-1650 nm	No pre-treatment	18	0.920	1.946	4.788	0.920	0.803	5.813	7.179	0.802
	Normalized	12	0.822	4.359	7.165	0.822	0.728	8.097	9.119	0.681
	1st derivate	11	0.909	2.235	5.131	0.909	0.884	5.007	14.719	0.170
	2nd derivate	4	0.434	13.914	12.802	0.434	0.376	15.844	12.107	0.438
	Baseline-extras	18	0.920	1.956	4.801	0.920	0.782	6.613	7.461	0.786
	SNV	15	0.885	2.823	5.766	0.885	0.832	6.141	7.060	0.809
	Detrend	17	0.915	2.0927	4.965	0.915	0.810	6.106	6.691	0.828
	MSC	13	0.811	4.636	7.389	0.811	0.635	10.426	10.027	0.615
	Smoothing	18	0.916	2.065	4.933	0.916	0.767	7.435	8.701	0.710
Rapeseed oils 350-1650 nm	No pre-treatment	10	0.954	1.0355	4.002	0.954	0.988	2.801	7.922	0.759
	Normalized	18	0.987	0.285	2.102	0.987	0.960	5.036	8.922	0.695
	1st derivate	7	0.955	1.006	3.945	0.955	0.990	2.947	5.672	0.876
	2nd derivate	8	0.960	0.898	3.728	0.960	0.918	4.992	7.250	0.798
	Baseline	11	0.956	0.893	3.689	0.956	0.958	4.359	7.740	0.808
	SNV	17	0.980	0.393	2.449	0.980	0.965	6.907	9.471	0.712
	Detrend	7	0.933	1.374	4.576	0.933	0.989	3.582	7.071	0.839
	MSC	20	0.989	0.206	1.774	0.989	0.878	7.753	9.034	0.738
	Smoothing	5	0.807	3.982	7.789	0.807	0.683	7.349	8.124	0.788
Rapeseed oils 500-1650 nm	No pre-treatment	18	0.983	0.379	2.423	0.983	0.935	0.870	8.446	0.727
	Normalized	15	0.964	0.794	3.505	0.964	0.772	4.216	7.841	0.764
	1st derivate	6	0.927	1.642	5.039	0.927	0.855	5.283	8.248	0.739
	2nd derivate	15	0.994	0.013	0.452	0.999	0.872	4.494	8.039	0.752
	Baseline	14	0.967	0.674	3.206	0.967	0.909	4.657	7.710	0.809
	SNV	17	0.973	0.545	2.882	0.973	1.027	2.073	9.980	0.680
	Detrend	8	0.892	2.220	5.816	0.892	0.887	4.985	7.278	0.830
	MSC	17	0.974	0.526	2.831	0.974	1.002	1.081	10.881	0.620
	Smoothing	18	0.976	0.477	2.696	0.976	0.970	3.527	9.830	0.690
Soybean oils 350-1650 nm	No pre-treatment	5	0.920	1.565	4.990	0.920	0.887	4.410	6.323	0.870
	Normalized	3	0.836	3.214	7.151	0.836	0.871	4.502	6.519	0.861
	1st derivate	7	0.860	2.753	6.619	0.860	0.873	4.442	5.650	0.896
	2nd derivate	9	0.833	3.284	7.228	0.833	0.766	4.993	7.539	0.815
	Baseline	5	0.926	1.454	4.810	0.926	0.858	3.980	5.702	0.894
	SNV	4	0.885	2.265	6.0003	0.885	0.862	4.507	6.745	0.852
	Detrend	4	0.894	2.081	5.754	0.894	0.949	3.237	5.035	0.917
	MSC	4	0.874	2.474	6.273	0.874	0.288	19.741	18.396	0.229
	Smoothing	5	0.913	1.711	5.218	0.913	0.909	3.684	5.188	0.912
Soybean oils 500-1650 nm	No pre-treatment	6	0.923	1.503	4.890	0.923	0.888	4.074	5.509	0.901
	Normalized	20	0.985	0.287	2.139	0.985	0.918	1.677	6.674	0.855
	1st derivate	19	0.860	2.753	6.619	0.860	0.873	4.442	5.650	0.896
	2nd derivate	6	0.964	0.695	3.332	0.964	0.919	2.507	4.771	0.926

	Baseline	20	0.997	0.044	0.845	0.997	0.921	3.118	5.938	0.885
	SNV	15	0.969	0.593	3.073	0.969	0.980	0.623	5.843	0.889
	Detrend	11	0.981	0.361	2.399	0.981	0.918	2.310	5.228	0.911
	MSC	5	0.845	3.051	6.968	0.845	0.564	14.599	13.741	0.386
	Smoothing	6	0.923	1.514	4.908	0.923	0.888	4.091	5.521	0.900
Sunflower oils 350-1650 nm	No pre-treatment	18	0.989	0.195	1.774	0.989	0.943	0.463	5.547	0.906
	Normalized	20	0.987	0.2223	1.898	0.987	0.877	1.518	6.608	0.867
	1st derivate	11	0.988	0.222	1.892	0.988	0.914	0.331	7.093	0.846
	2nd derivate	9	0.980	0.360	2.408	0.980	0.975	-1.287	9.310	0.736
	Baseline	10	0.943	1.049	4.1111	0.934	0.950	-0.620	5.262	0.915
	SNV	17	0.980	0.355	2.938	0.980	0.863	1.648	7.542	0.826
	Detrend	14	0.975	0.460	2.722	0.975	0.971	-0.887	7.025	0.849
	MSC	19	0.988	0.210	1.841	0.988	0.814	3.260	7.209	0.841
	Smoothing	11	0.949	0.939	3.890	0.949	0.942	-0.620	5.682	0.901
Sunflower oils 500-1650 nm	No pre-treatment	14	0.969	0.567	3.021	0.969	0.967	-0.889	6.805	0.859
	Normalized	17	0.974	0.481	2.784	0.974	0.921	-0.258	6.145	0.885
	1st derivate	13	0.997	0.047	0.878	0.997	0.924	-0.993	7.577	0.825
	2nd derivate	9	0.980	0.360	2.408	0.980	0.975	-1.287	9.310	0.736
	Baseline	14	0.975	0.452	2.698	0.975	0.997	-0.415	7.452	0.830
	SNV	9	0.889	2.061	5.760	0.889	1.000	-2.763	7.645	0.822
	Detrend	12	0.967	0.607	3.128	0.967	0.986	-0.929	6.270	0.880
	MSC	10	0.916	1.548	4.993	0.916	0.990	-2.143	7.158	0.844
	Smoothing	14	0.967	0.604	3.119	0.967	0.965	-0.959	6.834	0.857
